# Promoting physical activity among university students with a co-constructed program during Covid-19 pandemic

**DOI:** 10.1192/j.eurpsy.2022.1387

**Published:** 2022-09-01

**Authors:** A. Goncalves, M. Deshayes, C. Bernal, K. Korchi, M. Nogrette, B. Gisclard, E. Charbonnier

**Affiliations:** 1 APSY, Université De Nîmes, Nimes, France; 2 Projekt, Université De Nîmes, Nimes, France

**Keywords:** physical activity, co-construction, University student, interventional study

## Abstract

**Introduction:**

Since the beginning of the COVID-19 pandemic, sanitary context and e-learning has greatly modified students’ lifestyles. An increase of sedentary behaviors, a reduction in physical activity (PA) and a stronger tendency to move towards unhealthy diet have been demonstrated. Most of the research is largely descriptive and to date, no interventional studies have been conducted to prevent the deterioration of students’ health.

**Objectives:**

The objective of the present research aims to evaluate the effects of an intervention program on the lifestyle and psychological state of student. Its primary objective is to promote PA among students, to improve both physical condition and motivation to engage in physical activity for one’s health by promoting motivational levers. Its second objective is to reduce and/or prevent the deterioration of the health of university students.

**Methods:**

Students from University of Nîmes were recruited and randomly assigned to one of the two following conditions: an experimental group and a control group. The experimental group participated to an 8-weeks program of PA (co-constructed by users during design-based innovative workshops) whereas the control group did not. For each group, measures of PA, sedentary time, anthropometric data, sleep, physical condition and psychological variables (anxiety, depression, motivation, body appreciation, perceived control, well-being, …) were carried out before (T1: october 2021) and after (T2: December 2021) these 8-weeks in order to evaluate the benefits from the PA program.

**Results:**

These assessments were performed in October 2021 (T1) and December 2021 (T2).

**Conclusions:**

Data are still being collected and will be presented in April 2022.

**Disclosure:**

No significant relationships.

